# Prevalence of rheumatic regional pain syndromes in Latin-American indigenous groups: a census study based on COPCORD methodology and syndrome-specific diagnostic criteria

**DOI:** 10.1007/s10067-016-3188-y

**Published:** 2016-02-01

**Authors:** José Alvarez-Nemegyei, Ingris Peláez-Ballestas, Mario Goñi, Flor Julián-Santiago, Conrado García-García, Rosana Quintana, Adriana M. R. Silvestre, Imelda García-Olivera, Nora A. Mathern, Adalberto Loyola-Sanchez, Silvana Conti, Alvaro J. Sanabria, Bernardo A. Pons-Estel

**Affiliations:** Unidad de Investigación, Hospital Regional de Alta Especialidad de la Península de Yucatán, Secretaría de Salud, Calle 7 No. 433 por 20 y 22, Fracc, Altabrisa, C.P. 97130 Mérida, Yucatán Mexico; Rheumatology Unit, Hospital General de México “Eduardo Liceaga”, Secretaría de Salud, México, D.F. Mexico; Centro de Especialidades Médicas Ambulatorias de Rosario (CEMAR), Secretaría de Salud Pública, Municipalidad de Rosario, Santa Fe, Argentina; Unidad de Posgrado, UNAM, México, Mexico; Hospital Provincial de Rosario, Santa Fe, Argentina; Ministerio de Salud de la Provincia de Santa Fe, Rosario, Santa Fe Argentina; Hospital Regional de Alta Especialidad, Secretaría de Salud, Oaxaca, Mexico; IROF, Rosario, Argentina; School of Rehabilitation Science, McMaster University, Hamilton, Canada

**Keywords:** Soft tissue rheumatology, Rheumatic regional pain syndrome, Tendonitis, Bursitis, Epidemiology, Prevalence, Ethnicity

## Abstract

This study assessed the overall and specific prevalence of the main rheumatic regional pain syndromes (RRPS) in four Latin-American indigenous groups. A Community Oriented Program for Control of Rheumatic Diseases (COPCORD) methodology-based census study was performed in 4240 adults (participation rate: 78.88 %) in four indigenous groups: Chontal (Oaxaca, Mexico, *n* = 124), Mixteco (Oaxaca, Mexico; *n* = 937), Maya-Yucateco (Yucatán, Mexico; *n* = 1523), and Qom (Rosario, Argentina; *n* = 1656). Subjects with musculoskeletal pain were identified using a cross-cultural, validated COPCORD questionnaire administered by bilingual personnel, and reviewed by general practitioners or rheumatologists using standardized case definitions for the 12 most frequent RRPS. The overall prevalence of RRPS was confirmed in 239 cases (5.64 %, 95 % CI: 4.98–6.37). The prevalence in each group was Chontal *n* = 19 (15.32 %, 95 % CI: 10.03–22.69); Maya-Yucateco *n* = 165 (10.83 %, 95 % CI: 9.37–12.49); Qom *n* = 48 (2.90 %, 95 % CI: 2.19–3.82); and Mixteco *n* = 7 (0.75 %, 95 % CI: 0.36–1.53). In the whole sample, the syndrome-specific prevalence was rotator cuff tendinopathy: 1.98 % (95 % CI: 1.60–2.45); lateral epicondylalgia: 0.83 % (95 % CI: 0.59–1.15); medial epicondylalgia: 0.73 % (95 % CI: 0.52–1.04); biceps tendinopathy: 0.71 % (95 % CI: 0.50–1.01); anserine syndrome: 0.64 % (95 % CI: 0.44–0.92); inferior heel pain: 0.61 % (95 % CI: 0.42–0.90); trochanteric syndrome: 0.49 % (95 % CI: 0.25–0.64); de Quervain’s tendinopathy: 0.45 % (95 % CI: 0.29–0.70); trigger finger: 0.42 % (95 % CI: 0.27–0.67); carpal tunnel syndrome: 0.28 % (95 % CI: 0.16–0.49); Achilles tendinopathy (insertional): 0.12 % (95 % CI: 0.05–0.28); and Achilles tendinopathy (non-insertional): 0.07 % (95 % CI: 0.02–0.21). Leaving aside the comparison between Maya-Yucateco and Chontal groups (*p* = 0.18), we found significant differences (*p* < 0.001) in overall RRPS prevalence between the remaining pairs of indigenous groups. Syndrome-specific prevalences were also different between groups. Our findings support the hypothesis that overall RRPS prevalence and syndrome-specific prevalences are modulated by population-specific factors.

## Introduction

Rheumatic regional pain syndromes (RRPS), also known as soft tissue rheumatic syndromes and non-articular rheumatism, is a collective term for a group of clinical disorders characterized by pain and functional impairment in a specific region of the appendicular musculoskeletal (MSK) system, arising from an acute or chronic overuse injury of a para-articular structure, such as a tendon, ligament, bursa, or fascia. Some non-MSK disorders, such as entrapment neuropathies (e.g., carpal tunnel syndrome), are also categorized as RRPS because they have similar clinical manifestations and usually share etiologic pathways [1].

Although RRPS are among the most prevalent rheumatic diseases, their epidemiological impact has not been definitively established. There have been a number of studies in different target populations of the prevalence of some RRPS, such as shoulder pain and rotator cuff tendinopathy [2–5], epicondylalgia [6, 7], carpal tunnel syndrome [8, 9], pes anserinus bursitis [10], and trochanteric syndrome [11], but there was heterogeneity in case definitions. The overall prevalence of RRPS has been assessed in a series of open population studies, mostly in developing countries [12–28]. There is only one report on the individual prevalence of RRPS [25]. The overall prevalence of RRPS in these studies showed marked variations, ranging from 0.7 to 15.0 %, even though the screening tool was in all of them the Community Oriented Program for Control of Rheumatic Diseases (COPCORD) questionnaire [29]. It is unclear whether the extremely wide variation in prevalence was real, or a result of defective or heterogeneous case definition, because the diagnosis of RRPS was based on the clinical judgment of the surveying physician and did not rely on a standardized or validated set of diagnostic or classification criteria.

One nationwide, community-based study in Mexico that assessed the overall and syndrome-specific prevalence of RRPS using COPCORD screening and standardized definitions for RRPS case definitions [30] found an important variation in overall RRPS prevalence between different regions of the country, which was probably influenced by population-specific socioeconomic and/or biological factors [31, 32].

There is a lack of scientific information concerning the overall and syndrome-specific prevalence of RRPS in indigenous groups, including those from Latin America. In fact, there are only two COPCORD-based studies on that subject [18, 27]. Consequently, the aim of the current study was to evaluate and compare the overall and syndrome-specific prevalence of the most important RRPS in four different Latin-American adult-indigenous communities in Mexico and Argentina, using the COPCORD screening methodology and a set of validated or standardized diagnostic criteria for case definition.

## Material and methods

### Participants

The present study was planned to include all inhabitants aged 18 or older living in selected and officially representative communities of the following indigenous groups: Maya-Yucateco, Mixteco, and Chontal in Mexico and Qom (or Toba) in Argentina. For the Maya-Yucateco people, we selected the rural community of Chankom, located 123 km from Merida, the capital city of the Mexican Southeastern State of Yucatan. According to the 2010 Population National Census of Mexico, this community had 2242 adults. This Mayan Municipality lies between parallels 20°80′ and 20°39′ north latitude and meridians 88°28′ and 88°38′ west longitude; average height is 27 m above sea level. The terrain is flat with no hilly areas of relevance; soils are composed of jagged rocks. A warm humid climate with summer rains predominates in the region. Average annual temperature is 26.2 °C and average annual rainfall is 35.8 mm. The main economic activities in the municipality are farming and livestock (cattle) [33]. The Chontales and the Mixtecos are located in the Western Sierra Madre region at an altitude of 2340 m above sea level. The temperature in the region ranges between 10 and 14 °C and 24 and 26 °C, with the average rainfall of 700 mm per year. The selected Chontal community was San Carlos Yautepec, a very small rural village located at the Sierra Madre del Sur, with 128 adult inhabitants. The highland Mixteco people were represented by the rural community of San Antonio Huitepec, which has 951 adult inhabitants [34]. Both communities are located in the highland areas of the Southern Mexican State of Oaxaca and are 217 km apart. The Mayan Municipality of Chankom is located 1329 km away from the Chontal and the Mixteco communities included in this study [33, 34]. The selected Qom indigenous group lives in three districts of the City of Rosario, Santa Fe province, Argentina. According to a previous census performed by us, this community has 2157 adults. As 103 adults had participated in a study of cross-culturization of the COPCORD instrument, the number available for our study was 2054. According to official statistics of Mexico and Argentina, the selected indigenous communities can be categorized as having a high level of social deprivation because almost all of their inhabitants live below the poverty line [35].

### Study design

A map of the four selected indigenous communities was used for a systematic evaluation of every home in the community. Each home was approached by a team of researchers composed by a trained bilingual translator and a general practitioner; every person aged 18 or older was invited to participate in the study. In the specific case of Qom population, the field research team was composed by at least four participants: a trained bilingual translator, a coordinator, a medical or nursing student, and a specialist in internal medicine. Cases were selected after signing informed according to the three phases suggested in stage I of the COPCORD methodology [29]. The use of a cross-cultural, validated COPCORD questionnaire in the appropriate indigenous language [35] allowed the identification of subjects with MSK pain during the last 7 days (identified for study purposes as COPCORD-positive subjects) and inclusion of the subject in the subsequent stages of the survey. Subsequently, every subject with non-traumatic MSK pain localized in the extremities underwent a clinical examination by a general practitioner or a specialist in internal medicine (in the case of Qom population) or rheumatologist, either at home or at a community primary care facility. All indigenous groups from Oaxaca were evaluated by the same health professional team. COPCORD-positive subjects without RRPS were evaluated by a rheumatologist in order to provide a diagnosis according to international criteria for rheumatic diseases. The study period was from June 2011 to December 2012.

### RRPS case definition

Upper limb syndromes (apart from trigger finger) were defined according to the Southampton group criteria, whose validity and consistency have been established for epidemiological research [36, 37]. For RRPS of the lower limbs, we used an expert consensus for a specific case definition of trochanteric syndrome, pes anserinus bursitis, Achilles tendinopathy, and inferior heel pain, in addition to trigger finger; these criteria were previously used for a similar nationwide study of RRPS prevalence in Mexico [30]. For each specific RRPS, a criteria-based checklist was developed, and every subject with a final diagnosis of RRPS had to fulfill the diagnostic checklist for a specific syndrome. All participant general practitioners, specialist in internal medicine, and rheumatologists underwent training by a single expert to standardize the case definition methodology.

### Statistical analysis

Prevalence (%) with 95 % CI was determined for overall RRPS and for each of the 12 specific syndromes. The unpaired *t* test, one-way analysis of variance, chi-square test with Yates correction, or Fischer’s exact test was also used as appropriate for group comparisons. Analysis was performed using SPSS 20.0 (IBM Corp., Armonk, NY, USA) and Stata statistical software (Stata Corp., College Station, TX, USA). The significance level was set at 0.05.

### Ethical issues

The protocol was approved by the Ethics Committee on Research of the Hospital General de México (Mexico, City), the Research Committee of the Universidad Anáhuac-Mayab (Mérida, Mexico), the Research and Ethics Committee of the Secretaría de Salud, Municipalidad de Rosario Argentina, and the Santa Fe Provincial Bioethics Committee (Argentina). Approval for the study was obtained as required from the community and indigenous authorities of each participating group. After a detailed explanation of the study, each subject gave his/her consent in his/her own language to participate in the study. All subjects identified as having any rheumatic or non-rheumatic disease and not having access to medical care were advised to seek assistance according to their local health care system.

## Results

A total of 4240 subjects of 5375 eligible adults living in the four selected communities consented to participate in the survey (overall participation rate: 78.88 %). Group demographics, participation rates, and rate of subjects having MSK pain in the last 7 days (COPCORD-positive) are presented in Table 1. There was no gender difference in participation rates (*p* = 0.78). The Qom people were younger (*p* < 0.001) and had a lower prevalence of COPCORD-positive subjects compared with the other ethnic groups (*p* < 0.001).

**Table 1:** Sample size, participation rate, basic demographic features, and the prevalence of musculoskeletal pain in the last 7 days between the four studied indigenous groups

Indigenous group:	Participation		Female/male		Participant’s age (years)		MS pain in last 7 days		
	*n*	%	*n*	%	Mean ± D.S.	Range	*n*	%	95 % CI
Maya-Yucateco (*n* = 2442)	1523	62.36	917/606	60.2/39.8	45.2 ± 17.9	18–104	592	38.87	36.45–41.34
Mixteco (*n* = 951)	937	98.52	570/367	60.8/39.2	46.9 ± 20.1	17–97	432	46.10	42.92–49.31
Chontal (*n* = 128)	124	96.87	72/52	58.1/41.9	47.0 ± 18.1	19–89	51	41.13	32.86–49.93
Qom (*n* = 2054)	1656	80.62	1020/636	61.6/38.4	35.3 ± 13.9	18–105	471	28.44	26.32–30.66
Overall (*n* = 5375)	4240	78.88	2579/1661	60.8/39.2	41.8 ± 17.8	17–105	1546	36.55	35.11–38.01

*MS* musculoskeletal

Of 1546 COPCORD-positive subjects, 239 fulfilled the criteria for at least one RRPS; thus, the overall RRPS prevalence was 5.64 % (95 % CI: 4.98–6.37). In COPCORD-positive population, subjects with RRPS were older (49.4 ± 14.9 vs. 41.3 ± 17.8 years; *p* < 0.001) than those who did not have RRPS. On the other hand, no gender difference was found between subjects having (male: 35.9.8 %/female: 64.1 %) and not having (male: 39.4 %/female: 60.6 %; *p* = 0.32) RRPS as final diagnosis.

There were 309 RRPS identified; 45 subjects had >1 syndrome: 33 had two; seven had three; three had four; one subject had six; and another had eight different syndromes.

There were some differences in the prevalence of RRPS per indigenous groups. The prevalence of RRPS Chontal and Maya-Yucateco groups did not differ between them, but their prevalence was higher than those of the Mixteco and Qom groups. Lastly, the overall prevalence of RRPS in the two latter groups was significantly different (Table 2).

**Table 2:** Comparison^a^ of the RRPS overall prevalence between the four studied indigenous groups

Ethnical group:	Subjects with RRPS	Prevalence (%)	95 % CI^b^
Chontal (*n* = 124)	19	15.32	10.03–22.69
Maya-Yucateco (*n* = 1523)	165	10.83	9.37–12.49
Qom (*n* = 1656)	48	2.90	2.19–3.82
Mixteco (*n* = 937)	7	0.75	0.36–1.53

^a^ *X* ^2^ results: Chontal vs. Maya: *p* = 0.18; Chontal vs. Qom: *p* < 0.0001; Chontal vs. Mixteco: *p* < 0.0001; Mava vs. Qom: *p* < 0.0001; Maya vs. Mixteco: *p* < 0.0001; Qom vs. Mixteco: *p* < 0.0001

^b^Prevalence’s 95 % confidence interval

The prevalence of each of the 12 specific RRPS is shown in Table 3. Shoulder rotator cuff tendinopathy was the most frequent syndrome, followed by medial and lateral epicondylalgia, shoulder bicipital tendinopathy, pes anserinus bursitis, and inferior heel pain. The most prevalent of the lower limb syndromes was pes anserinus bursitis. Excepting lateral or medial epicondylalgia, and biceps tendinopathy, there was a trend for a higher prevalence for the rest of RRPS in women, but analysis of the 95 % CIs did not show significant differences.

**Table 3:** Overall and gender specific prevalence^a^ for every one of the 12 studied RRPS in the whole surveyed population

Syndrome:	Overall *n* = 4240		Men *n* = 1661		Women *n* = 2579	
	Cases *n*	Prevalence % (95 % CI)	Cases *n*	Prevalence % (95 % CI)	Cases *n*	Prevalence % (95 % CI)
Rotator cuff tendinopathy	84	1.98 (1.60–2.45)	30	1.81 (1.27–2.57)	54	2.09 (1.61–2.72)
Biceps tendinopathy	30	0.71 (0.50–1.01)	12	0.72 (0.41–1.26)	18	0.70 (0.44–1.10)
Lateral epicondylalgia	35	0.83 (0.59–1.15)	18	1.08 (0.69–1.71)	17	0.66 (0.41–1.05)
Medial epicondylalgia	31	0.73 (0.52–1.04)	13	0.78 (0.46–1.33)	18	0.70 (0.44–1.10)
Carpal tunnel syndrome	12	0.28 (0.16–0.49)	2	0.12 (0.03–0.44)	10	0.39 (0.21–0.71)
de Quervain’s tendinopathy	19	0.45 (0.29–0.70)	3	0.18 (0.06–0.53)	16	0.62 (0.38–1.01)
Trigger finger	18	0.42 (0.27–0.67)	4	0.24 (0.09–0.62)	14	0.54 (0.32–0.91)
Trochanteric syndrome	17	0.49 (0.25–0.64)	4	0.24 (0.09–0.629	13	0.50 (0.29–0.86)
Anserine syndrome	27	0.64 (0.44–0.92	9	0.54 (0.29–1.03)	18	0.70 (0.44–1.10)
Achilles tendinopathy (non-insertional)	3	0.07 (0.02–0.21)	0	–	3	0.12 (0.04–0.34)
Achilles tendinopathy (insertional)	5	0.12 (0.05–0.28)	1	0.06 (0.01–0.34)	4	0.16 (0.06–0.40)
Inferior heel pain	26	0.61 (0.42–0.90)	8	0.48 (0.24–0.95)	18	0.70 (0.44–1.10)

^a^95 % confidence interval between brackets

Comparative analysis of each RRPS across the four indigenous groups (Table 4) showed that rotator cuff tendinopathy was consistently the most frequent syndrome, followed by shoulder biceps tendinopathy, lateral epicondylalgia, and pes anserinus bursitis in each indigenous group. Chontal subjects showed a tendency for a higher relative frequency of lower limb RRPS (44 %) compared with Maya-Yucateco (34 %), Qom (20 %), or Mixteco (15 %) subjects, but there were no significant differences (*p* = 0.35).

**Table 4:** Comparative prevalence^a^ of the 12 assessed RRPS between the four studied indigenous groups

Syndrome:	Ethnical group							
	Qom (*n* = 1656)		Mixteco (*n* = 937)		Chontal (*n* = 124)		Maya-Yucateco (*n* = 1523)	
	Cases *n*	Prevalence % (95 % CI)	Cases *n*	Prevalence % (95 % CI)	Cases *n*	Prevalence % (95 % CI)	Cases *n*	Prevalence % (95 % CI)
Rotator cuff tendinopathy	15	0.91 (0.55–1.49)	4	0.43 (0.17–1.09)	10	8.06 (4.44–14.21)	55	3.61 (2.78–4.67)
Biceps tendinopathy	11	0.66 (0.37–1.19)	1	0.11 (0.02–0.60)	4	3.23 (1.26–8.00)	14	0.92 (0.55–1.54)
Lateral epicondylalgia	9	0.54 (0.29–1.03)	0	–	1	0.81 (0.14–4.43)	25	1.64 (1.11–2.41)
Medial epicondylalgia	2	0.12 (0.03–0.44)	1	0.11 (0.02–0.60)	3	2.42 (0.83–6.87)	25	1.64 (1.11–2.41)
Carpal tunnel syndrome	3	0.18 (0.06–0.53)	0	–	4	3.23 (1.26–8.00)	5	0.33 (0.14–0.77)
de Quervain’s tendinopathy	2	0.12 (0.03–0.44)	0	–	4	3.23 (1.26–8.00)	13	0.85 (0.50–1.45)
Trigger finger	2	0.12 (0.03–0.44)	0	–	0	–	16	1.05 (0.65–1.70)
Trochanteric syndrome	4	0.24 (0.09–0.62)	0	–	2	1.61 (0.44–5.69)	11	0.72 (0.40–1.29)
Anserine syndrome	2	0.12 (0.03–0.44)	0	–	5	4.03 (1.73–9.09)	20	1.31 (0.85–2.02)
Achilles tendinopathy (non-insertional)	0	–	0	–	1	0.81 (0.14–4.43)	2	0.13 (0.04–0.48)
Achilles tendinopathy (insertional)	0	–	0	–	5	4.03 (1.73–9.09)	0	–
Inferior heel pain	4	0.24 (0.09–0.62)	1	0.11 (0.02–0.60)	7	5.65 (2.76–11.19)	14	0.92 (0.55–1.54)

^a^95 % confidence interval between brackets

## Discussion

The present study shows that the overall RRPS prevalence for the four studied Latin-American indigenous communities was 5.64 %. There were marked differences in overall and syndrome-specific RRPS prevalences between the Latin-American indigenous populations. A noteworthy finding was that despite these differences, rotator cuff tendinopathy was consistently the most frequent RRPS in each indigenous group.

The overall RRPS prevalence reported here was within the range (0.7–15.0 %) reported in other COPCORD-based studies performed worldwide [12–28] and is similar to the overall RRPS prevalence reported in a Mexican study that used the same screening and case definition methodology [30]. The concordance between two studies indicate that the wide variability in RRPS prevalence previously reported could be related to a lack of uniformity in RRPS case definition. However, the Mexican study also found differences in RRPS prevalence between the four geographical regions of the country [30], and a COPCORD-based study in Guatemala [27] found differences in RRPS prevalence between the Mestizo and indigenous populations. This supports the hypothesis that variations in RRPS prevalence between populations are real and may be explained, at least partially, by biological (i.e., polymorphic variants of structural tendinous proteins), socioeconomic (burden of biomechanical job demand, and type of job), or ethnic (i.e., biomechanical demand derived from physical recreational activities) factors. The results of the current study, where marked differences were found in overall RRPS prevalence between most of the studied indigenous groups, provide further support for this hypothesis.

In most indigenous groups, the overall and syndrome-specific prevalence of RRPS remain relatively unexplored issues. The prevalence of RRPS in aboriginal or indigenous groups has been specifically examined using COPCORD screening methodology in only two studies. Minaur et al. reported a prevalence of 7.4 % for “soft tissue pain” in Australian aboriginals [18]. Obregón-Ponce et al. found a prevalence of “soft tissue rheumatisms” in the Mayan-Kaqchiquel group of 2.3 % [27]. The difference between our RRPS prevalence and these two studies may partly arise because the previous studies relied on individual physicians’ diagnosis of RRPS, instead of validated uniform case definitions. The differences in screening methodology and case definitions between this report and non-COPCORD-based studies of the prevalence of some specific RRPS, such as painful shoulder [2], rotator cuff tendinopathy [3–5], epicondylalgia [6, 7], carpal tunnel syndrome [8, 9], pes anserinus bursitis [10], and trochanteric syndrome [11], may have contributed to the differences in prevalence rates obtained in the current study and in previous studies.

The prevalence of the main specific RRPS using COPCORD methodology has only been explored in two reports. Sandoughi et al. [25] investigated the specific prevalence of six RRPS: rotator cuff tendinopathy, lateral and medial epicondylalgia, carpal tunnel syndrome, de Quervain’s tendinopathy, and trigger finger, diagnosed by individual clinical judgment, in the Southeastern part of Iran. The prevalences were similar to our findings for only two of the six studied RRPS: lateral epicondylalgia and de Quervain’s tendinopathy. They reported a relatively higher prevalence of carpal tunnel syndrome and a markedly lower prevalence of trigger finger, medial epicondylalgia, and rotator cuff tendinopathy compared with our study. In the nationwide Mexican study, where the same 12 individual RRPS were assessed with the same screening methodology and case definition, only the prevalences of trigger finger and inferior heel pain were similar to our findings [30]. There was a consistently higher prevalence of nine of the 10 remaining syndromes (not carpal tunnel syndrome) in our indigenous populations compared with the results of the Mexican study, which was based on the Mestizo population. This lends support to the hypothesis that overall and specific RRPS vary according to the population and may be influenced by inherent factors specific for each population.

A major limitation of this report is the difference in numbers of the studied indigenous groups. However, the census approach, use of an established method of screening for epidemiological studies in rheumatology (COPCORD), use of a cross-cultured screening instrument applied by trained bilingual staff, use of standardized case definitions, and a 78.88 % overall participation rate can be considered as major strengths of our study.

In conclusion, this study suggests that there are differences in the prevalence of overall and syndrome-specific RRPS between ethnic populations. Further multicenter studies, with adjustment for genetic, cultural, and socioeconomic factors are necessary to further investigate the reasons for this variability.
